# Serum inflammation and oxidative DNA damage amelioration in cocks-fed supplemental *Vernonia amygdalina* and zinc in aflatoxin B_1_ contaminated diets

**DOI:** 10.1093/tas/txad113

**Published:** 2023-09-16

**Authors:** Olumuyiwa J Olarotimi, Francis A Gbore, Olugbenga D Oloruntola, Olatunji A Jimoh

**Affiliations:** Department of Animal Science, Faculty of Agriculture, Adekunle Ajasin University, Akungba-Akoko, 342111 Ondo State, Nigeria; Department of Animal Science, Faculty of Agriculture, Adekunle Ajasin University, Akungba-Akoko, 342111 Ondo State, Nigeria; Department of Animal Science, Faculty of Agriculture, Adekunle Ajasin University, Akungba-Akoko, 342111 Ondo State, Nigeria; Department of Agricultural Technology, The Federal Polytechnic, Ado-Ekiti, 360231 Ekiti State, Nigeria

**Keywords:** antioxidant, blood, cytokines, mycotoxin, phytogenics, roosters

## Abstract

The objective of the study was to assess the comparative effects of *Vernonia amygdalina* leaf meal (**VALM**) and zinc (**Zn**) on the serum proinflammatory and anti-inflammatory cytokines as well as DNA damage of cocks-fed aflatoxin B_1_ (**AFB**_**1**_) contaminated diets. A total of 250 sexually mature Isa White cocks of 24 weeks old were randomly distributed into five groups (treatments) with each containing 50 birds, which was replicated five times with 10 birds per replicate. Cocks in group A were fed basal diet only, group B was fed basal diet contaminated with 1 mg AFB_1_/kg diet, group C received diet B (basal + 1 mg/kg AFB_1_) with 50 mg/kg Zn, group D was fed diet B with 2.5 g/kg VALM, and group E received diet B with 5.0 g/kg VALM, respectively. Feed and water were supplied *ad libitum* with fresh feed added to the feed troughs at 6:00 a.m. and 6:00 p.m., respectively. While serum tumor necrosis factor-alpha (**TNF-α**), interleukin 1 beta (**IL-1β**), 8-hydroxy-2ʹ-deoxyguanosine (**8-OHdG**), and nuclear factor kappa B (**NF-κB**) were significantly (*P* < 0.05) elevated among the cocks on diet B, significant (*P* < 0.05) reductions were recorded among cocks on diets C, D, and E. Conversely, birds in group B had significant (*P* < 0.05) depression in serum interleukin 4 (**IL-4**) and interleukin 10 (**IL-10**) while improvements (*P* < 0.05) were recorded among cocks in groups C, D, and E, respectively. Therefore, the inclusion of VALM offset the adverse physiological effects of AFB_1_ observed among group B birds. The effects were comparable with the results presented by the cocksfed diet containing Zn.

## INTRODUCTION

Mycotoxin contamination of food and feed is a global challenge leading to serious health issues in both humans and animals. Aflatoxins, which are generated by *Aspergillus flavus* and *Aspergillus parasiticus* and present a risk to the health of consumers, are critical food toxicants in subtropical and tropical areas (Hatipoglu and Keskin, 2022). Aflatoxin B_1_ (**AFB**_**1**_), regarded as a group 1 carcinogen, is nephrotoxic, mutagenic, immunotoxic, and teratogenic (Kumar et al., 2017). AFB_1_ readily contaminates raw feed ingredients, such as soy beans, groundnuts, millet, sorghum, maize, and wheat, due to the prevailing field and storage environmental factors that make these products susceptible to fungus infections. Only a small dose of AFB_1_ in feed ingredients is necessary to cause AFB_1_-induced health burdens in animals. Also, products such as eggs, milk, milk products, and meat from animals-fed AFB_1_-contaminated feed constitute a very great hazard to consumers, as AFB_1_-transformation products are easily transmitted (Fratamico et al., 2008).

Aflatoxicosis is a disease induced by aflatoxin ingestion, and it can be fatal (Mahato et al., 2019). In poultry, hens-fed aflatoxin-containing diets were found to have lower performance indicators such as laying efficiency and feed intake, as well as an undermined health status as evidenced by declined vaccination efficiency, diminished resistance to diseases, and subsequently greater mortality rates (Kassaw et al., 2022). Aflatoxins, too, play important functions in the regulation of cytokines and chemokines. Cytokines and chemokines are powerful signaling substances that play an important role in modulating intercellular communication in live cells (Ma et al., 2021). Both inflammatory and anti-inflammatory cytokines influence the immune response in both healthy and disease states. Nevertheless, the ability of cytokines to perform inflammatory, anti-inflammatory, or both roles is reliant on the specific local environment at the time (Chen et al., 2017). As a result, aflatoxins have been found to create a conducive atmosphere that adversely affects animal health by hampering certain anti-inflammatory cytokines as well as triggering some proinflammatory cytokines (Hatipoglu and Keskin, 2022; Popescu et al., 2022). Furthermore, AFB_1_ has previously been linked to oxidative DNA damage caused by the production of reactive oxygen species (**ROS**) in cells. Cellular ROS production indirectly assaults membrane phospholipids and emits different aldehydes that are carcinogenic in nature (Juan et al., 2021). AFB_1_ has been shown to enhance serum DNA damage indicators such as 8-hydroxy-2ʹ-deoxyguanosine (**8-OHdG**; AbuArrah et al., 2021) and nuclear factor kappa B (**NF-kB**; Pauletto et al., 2020). It midwifes the damage of DNA and various cellular proteins through the formation of AFB1-8,9 epoxide (**AFBO**), which binds to DNA to produce AFB_1_-DNA adducts which leads to mutations and carcinogenesis (Feng et al., 2016). It also disrupts protein and lipid metabolism by stimulating the production of ROS (Li et al., 2022). AFB_1_ was observed to significantly increase interleukin 6 (IL-6), interleukin 1 beta (**IL-1β**), and tumor necrosis factor-alpha (**TNF-α**) serum concentrations in another investigation (Cheng et al., 2023). Similarly, Rajput et al. (2021) reported blood elevations of IFN-γ, TNF-α, and other cytokines in broiler hens exposed to AFB_1_. Serum cytokine levels have traditionally been used as biomarkers in toxicology investigations to highlight inflammation and immune responses in tissue damage.

However, the use of natural antioxidants and immunosuppressive agents has come to light as one strategy to combat AFB_1_ toxicity. Plants are known for their rich phytochemical composition (Olarotimi et al., 2022). *Vernonia amygdalina* is among such plants with rich bioactive components which include flavonoids, alkaloids, terpenoids, and antioxidants like polyphenols (Adedapo et al., 2014). An essential trace element called zinc (**Zn**) is also crucial for maintaining DNA stability, redox balance, and immune system health (Bonaventura et al., 2015; Prasad et al., 2022). Zn supplementation has been researched for its potential to reduce oxidative stress and enhance immune response in poultry in the context of AFB_1_ toxicity (Hidayat et al., 2020; Hou et al., 2022). Therefore, the focus of this study is to investigate the potential therapeutic effects of *V. amygdalina* leaf meal (**VALM**) and Zn supplementation on serum DNA damage, proinflammatory cytokines, and anti-inflammatory cytokines in cocks-fed diets contaminated with AFB_1_. For the purpose of creating practical and effective strategies to protect poultry health and productivity in AFB_1_-contaminated environments, it is imperative to comprehend the additive effects of these therapies.

## MATERIALS AND METHODS

### Site, Materials, and Diets

The study was conducted at the Poultry Unit, Adekunle Ajasin University, Nigeria. The *V. amygdalina* leaves were sourced from the University’s Teaching and Research Farm and identified by an ethnobotanist at the University’s herbarium. A bottle of feed grade Zn powder was acquired from the institution’s agrochemical store. The leaves were properly washed to free them from any extraneous substances. They were left drained and air-dried for 7 days. The leaves were then milled into VALM to be included in the birds’ diets.

AFB_1_ was prepared by culturing *Aspergillus flavus* on maize grits and quantified for AFB_1_ and other Aspergillus mycotoxins as described by Mgbeahuruike et al. (2018). A basal diet containing 2,480 kcal/kg metabolizable energy, 15.03% crude protein, 1.13% calcium, 0.48% phosphorus, 1.10% lysine, 0.47% methionine, and 5.75% crude fiber was prepared. The diet was divided into five equal parts. A batch was labeled as the control diet (diet A). The remaining four batches were contaminated with AFB_1_ by including 6.25 g of the cultured maize in 1 kg of feed to give 1 mg/kg AFB_1_ concentration. To prepare experimental diets containing the target 1 mg/kg AFB_1_ concentration, 100 g of the cultured maize was included in 1 kg of feed, thoroughly mixed, and quantified for AFB_1_ concentration. The result of the analysis was 16 mg/kg AFB_1_ concentration. Therefore, this was used to calculate the amount of the cultured maize to be included in 1 kg of feed to give 1 mg/kg AFB_1_ concentration, thus:


$$\begin{array}{l} 100\;g\;cultured\;maize\;\equiv 16mg/kgAFB_{1} \end{array}$$ (1)

$$\begin{array}{l} Xgculturedmaize\;\equiv \;1\;mg/kgAFB_{1} \end{array}$$

$$\begin{array}{l} X=\frac{100g\times 1mg/kg}{16mg/kg}=6.25g \end{array}$$

Therefore, 6.25 g of the cultured maize was included and thoroughly mixed in 1 kg of feed. A sample of this mixture was quantified for AFB_1_ concentration, and the result was 1.02 mg/kg. A batch of the AFB_1_-contaminated lot was labeled as diet B. The remaining three batches were further reconstituted by the inclusion of 50 mg/kg Zn, 2.5 g/kg VALM, and 5.0 g/kg VALM representing diets C, D, and E, respectively.

### Experimental Animals and Procedures

A total of 250 sexually mature Isa White cocks 24 weeks old were supplied by the University’s farm. The cocks were raised in battery cages and managed under a strict biosecurity system. They were evenly assigned over the five experimental diets. Each treatment was in five replicates of 10 cocks each. They were given experimental diets and clean water *ad libitum* for the entire 12 weeks of the study. At the end of the field trial, blood samples from five cocks per replicate (i.e., 25 samples per treatment) were collected in clean bottles free of any form of anticoagulant and were correctly labeled to collect blood. The samples were left tilted in the rack for 15 min before being spun for 5 min at 5,000 × *g*, and a clean supernatant liquid was subsequently harvested and stored at −4 °F. The experimental procedures were in compliance with the guidelines for the Care and Use of Laboratory Animals. The experimental protocol was as approved by the institution’s Animal Research Ethics Committee. Ethics Reference No: AAUA/FA/ANS/4755/2023.

### Determination of Serum Cytokine Levels and DNA Damage

The serum samples were used for determination of TNF-α, IL-1β, interleukin 4 (**IL-4**), and interleukin 10 (**IL-10**) using CUSABIO commercial Chicken ELISA Kits while the concentrations of 8-OHdG and NF-κB were equally assayed using MyBioSource Inc. Chicken ELISA Kit.

### Statistical Analysis

Data collected from the study were analyzed statistically. They were subjected to one-way analysis of variance from General Linear Model for completely randomized design with the model: ϒ*xn* = μ + *αx* + β*xn* used. Where ϒ*xn* = any of the response variables; μ = the overall mean; *αx* = effect of the *x*th treatment (ϒ = diets A, B, C, D, and E) and β*xn* = random error due to experimentation. GraphPad Prism, software version 6.01 (2012) was used for the analysis. Tukey honestly significant difference of the same package was used for the post hoc analysis where there was a noticeable significant difference at *P* < 0.05.

## RESULTS

### Effects of AFB_1_ With or Without *V. amygdalina* and Zn on Serum Proinflammatory Cytokines

#### Serum interleukin 1beta (IL-1β).

**Figure 1.**
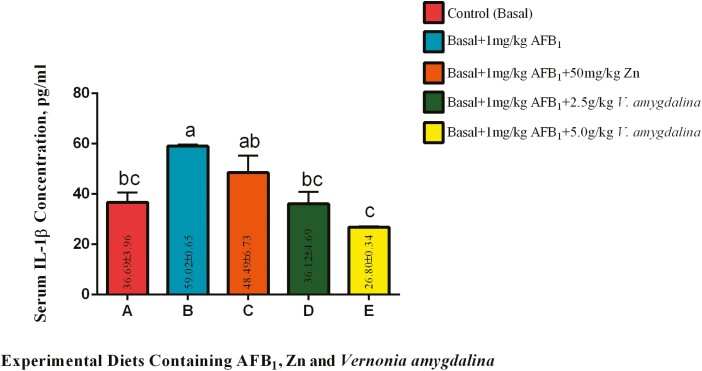
Effects of AFB_1_, Zn, and *Vernonia amygdalina* on serum IL-1β. Abbreviations: Zinc: Zn; AFB_1_: aflatoxin B_1_; IL-1β: interleukin 1beta.

The results of serum IL-1β concentrations of cocks fed 1 mg/kg AFB_1_ without organic or inorganic antioxidants and with varying inclusions of VALM and Zn are shown on Figure 1. The serum concentrations of IL-1β of cocks on diet containing 1 mg/kg AFB_1_ (diet B) was significantly (*P* < 0.05) increased when compared with birds on the control diet. However, a nonsignificant (*P* > 0.05) decrease in this parameter was recorded among the cocks-fed diet containing 50 g Zn/kg diet when compared with the value recorded among the birds-fed diets B and the control. The value recorded was statistically (*P* > 0.05) comparable with the mean value from the control birds. Furthermore, inclusion of 2.50 g VALM/kg diet fed to cocks in group D brought about a significant (*P* < 0.05) reduction in serum IL-1β concentration when compared with the value recorded among group B birds. The result of the inclusion of VALM at this level was not different (*P* > 0.05) from the values presented by the control cocks. It was further noted that the serum IL-1β concentration recorded among cocks-fed diet containing 5.0 g VALM/kg was significantly (*P* < 0.05) compared to the values recorded among the cocks on groups B and C, respectively, although statistically similar (P *>* 0.05) to the means recorded among the birds on groups A and D, respectively.

#### Serum tumor necrosis factor alpha (TNF-α).

**Figure 2.**
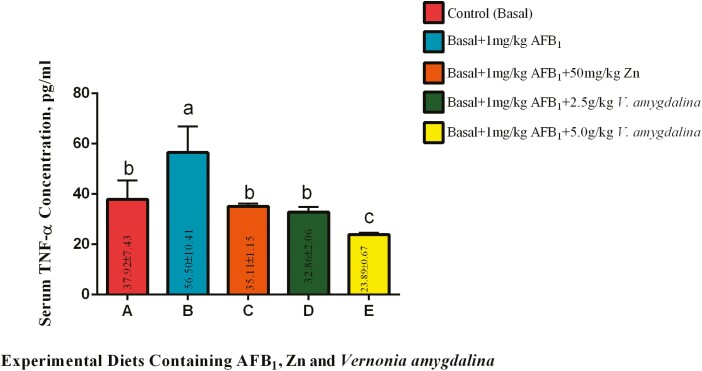
Effects of AFB_1_, Zn, and *Vernonia amygdalina* on serum tumor necrosis factor alpha TNF-α. Abbreviations: Zinc: Zn; AFB_1_: aflatoxin B_1_; TNF-α: tumor necrosis factor alpha.

Similar observations to Figure 1 were recorded for the serum TNF-α concentration (Figure 2) of the experimental birds. Dietary inclusion of 1 mg AFB1/kg diet significantly (*P* < 0.05) increased the concentration of the studied parameter among group B birds when compared with those on the control diet. Inclusions of Zn and 2.50 g VALM/kg diet in the aflatoxin-contaminated diet significantly (*P* < 0.05) reduced the serum concentrations of TNF-α among groups C and D, respectively, when compared with the cocks in group B. The outcome recorded in groups C and D were statistically (*P* > 0.05) similar to that of the control birds. Doubling the inclusion rate of VALM as observed among cocks in group E led to a further reduction (*P* < 0.05) in the serum concentrations of TNF-α. The result among the birds on diet E was a significant (*P* < 0.05) improvement when compared with results from groups A, C, and D, respectively.

### Effects of AFB_1_ With or Without *V. amygdalina* and Zn on Serum Anti-Inflammatory Cytokines

#### Serum interleukin 4 (IL-4).

**Figure 3.**
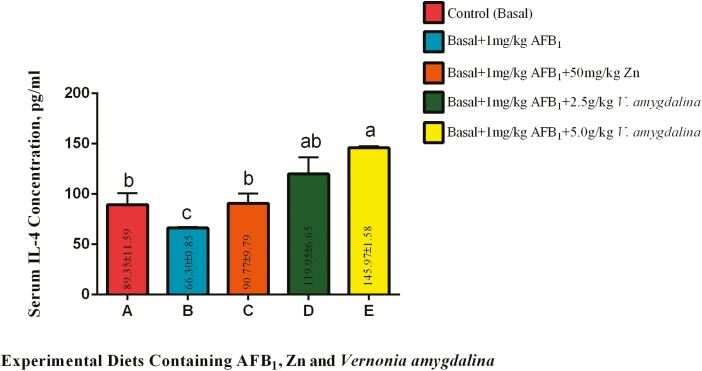
Effects of AFB_1_, Zn, and *Vernonia amygdalina* on serum IL-4. Abbreviations: Zinc: Zn; AFB_1_: aflatoxin B_1_; IL-4: interleukin-4.

The result of serum IL-4 concentrations of cocks-fed diets containing 1 mg/kg AFB_1_ without organic or inorganic antioxidants and with varying inclusions of VALM and Zn are shown in Figure 3. The least significant (*P* < 0.05) serum IL-4 concentrations were observed among the cocks on the diet containing 1 mg/kg AFB_1_ without antioxidant (diet B). The result was lower than the means recorded among the control birds when compared. However, there were significant (*P* < 0.05) enhancements in the serum IL-4 concentration among the birds in group C when compared with those in group B. These were the birds-fed diet containing AFB_1_ with Zn as a source of inorganic antioxidant. Furthermore, inclusions of 2.50 and 5.00 g/kg VALM significantly (*P* < 0.05) brought about increase in the serum IL-4 concentration of cocks in groups D and E, respectively when compared with birds in group B. It was also observed that better means were recorded among the birds in groups D (*P* > 0.05) and E (*P* < 0.05), respectively, when compared with the birds in group C. Doubling the inclusions of VALM, as the case in diets E, increased the serum concentrations (*P* > 0.05) of the parameter when compared with the values in diets D.

#### Interleukin 10 (IL-10).

**Figure 4.**
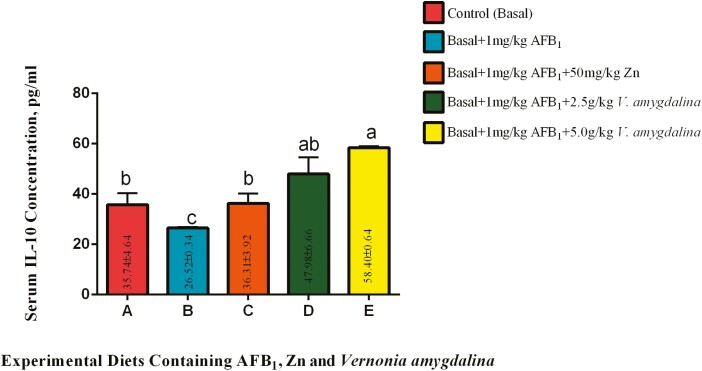
Effects of AFB_1_, Zn, and *Vernonia amygdalina* on serum IL-10. Abbreviations: Zinc: Zn; AFB_1_: aflatoxin B_1_; IL-10: interleukin-10.

The effects of AFB_1_ without varying inclusions of VALM and Zn are shown in Figure 4. There was a significant (*P* < 0.05) depression in serum IL-10 concentration among the birds-fed 1 mg/kg AFB_1_ diet when compared with the control birds. Inclusion of Zn in the diet containing 1 mg/kg AFB_1_ significantly (*P* < 0.05) enhanced the serum IL-10 concentration. Similarly, inclusions of 2.50 and 5.00 g/kg VALM significantly (*P* < 0.05) increased the serum concentration of IL-10 when compared with birds on diet B. Doubling the inclusion of VALM from 2.50 to 5.00 g/kg brought about a significant (*P* < 0.05) increase in serum IL-10 concentration when compared with birds on diet C.

### Effects of AFB_1_ With or Without *V. amygdalina* and Zn on DNA Damage

#### Serum 8-hydroxy-2’-deoxyguanosine (8-OHdG).

**Figure 5.**
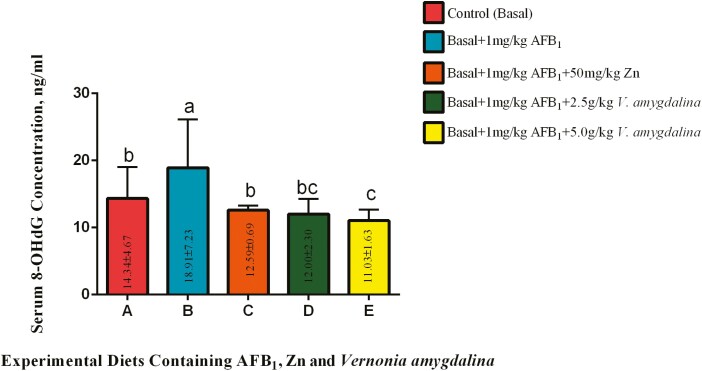
Effects of AFB_1_, Zn, and *Vernonia amygdalina* on serum 8-OHdG. Abbreviations: Zinc: Zn; AFB_1_: aflatoxin B_1_; 8-OHdG: 8-hydroxy-2’-deoxyguanosine.

 The results in Figure 5 highlight the effects of AFB1 with or without VALM and Zn on serum 8-OHdG. From the present experiment, inclusions of 1 mg/kg AFB_1_ in cock diet without further fortification with antioxidants significantly (*P* < 0.05) increased the serum 8-OHdG concentration of cocks in group B as compared with the birds on the control diet. Among the cocks in group C, inclusion of Zn significantly (*P* < 0.05) lowered the serum levels of the two mentioned parameters when compared with the birds on diet B. The Zn inclusion was able to bring down the elevated concentration of the parameter to a comparable state with the birds on the control diet. Likewise, inclusions of 2.50 g/kg VALM in this study significantly (*P* < 0.05) lowered the serum 8-OHdG concentration although with no significant (*P* > 0.05) difference when compared with the performance of the inorganic antioxidant. The inclusion of 5.0 g/kg VALM in the diet-fed cocks in group E did not only reduce (P *<* 0.05) the serum 8-OHdG concentration of the animals in comparison with group B birds but also lowered the parameters (*P* < 0.05) beyond what were obtainable among the cocks in diets A and C, respectively. However, there were statistical similarities (*P* > 0.05) in the serum 8-OHdG concentration among the birds in groups A, C, and D.

#### Serum nuclear factor kappa B (NF-kB).

**Figure 6.**
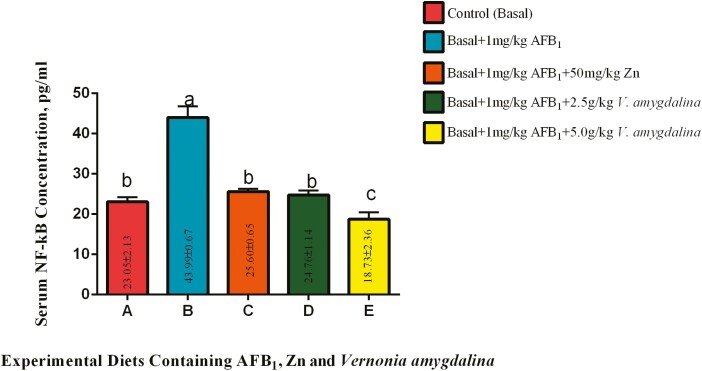
Effects of AFB_1_, Zn, and *Vernonia amygdalina* on serum NF-kB. Abbreviations: Zinc: Zn; AFB_1_: aflatoxin B_1_; NF-kB: nuclear factor Kappa B.

 The effects of AFB_1_, Zn, and VALM on serum NF-kB are shown in Figure 6. Inclusion of 1 mg/kg AFB_1_ in the diet (diet B) promoted the elevation of serum NF-kB concentration significantly (*P* < 0.05) among the cocks fed when compared with those on the control diet. However, inclusions of Zn and 2.50 g/kg VALM significantly (*P* < 0.05) brought down the serum concentration of this parameter among the birds on diets C and D when compared with cocks on diet B. The serum concentration of NF-kB among the on diets C and D were statistically similar (*P* > 0.05) to the means recorded among the control birds. Furthermore, a higher inclusion of VALM (5.00 g/kg) among the cocks on diet E significantly (*P* < 0.05) further decreased the serum concentration of the parameter when compared with birds from all other experimental diets.

## DISCUSSION

### Effects of AFB_1_ With or Without *V. amygdalina* and Zn on Serum Proinflammatory Cytokines and DNA Damage

In line with earlier studies (Bruneau et al., 2012; Hou et al., 2022) showing that exposure to AFB_1_ can cause an inflammatory response in different animal species, including poultry, the significant increase in serum IL-1β concentrations recorded in cocks exposed to 1mg/kg AFB_1_ (group B) compared with the control group supports the findings of Qian et al. (2014) and Li et al. (2014). According to Hatipoglu and Keskin (2022), AFB_1_ is known to impair immunological function and encourage the production of pro-inflammatory cytokines such IL-1β. However, the nonsignificant reduction in IL-1β levels seen in cocks on a diet containing 50 g Zn/kg diet (group C) as compared with group B is consistent with research on the protective effects of Zn against oxidative stress and inflammation (Bonaventura et al., 2015; Zhang et al., 2023). The trend seen in group C cocks may be explained by Zn, which is recognized for its immunomodulatory qualities and can control the production of proinflammatory cytokines (Akbari et al., 2018; Hidayat et al., 2020). Furthermore, VALM’s reported anti-inflammatory and immunomodulatory properties (Nimiya et al., 2016; Jin et al., 2021) are consistent with the significant decrease in serum IL-1β concentration caused by its inclusion in group D at 2.50 g/kg (compared to group B). The phytochemical makeup of VALM, which includes polyphenols and antioxidants, may play a role in its capacity to reduce inflammation brought on by AFB_1_. Further demonstrating the dose-dependent nature of VALM’s effect on IL-1β levels is a further significant decrease in IL-1β concentration seen in cocks fed diets containing 5.0 g VALM/kg (group E) compared to groups B and C. Other phytochemical therapies have also been demonstrated to have similar dose-dependent benefits (Adedapo et al., 2014; Olarotimi et al., 2022).

The increase in TNF-α in the cocks-fed diets containing 1 mg AFB_1_/kg as compared to the control group is similar to the findings for IL-1β, and it is consistent with earlier studies that have indicated that AFB_1_ can trigger proinflammatory cytokines, including TNF-α (Qian et al., 2014). This demonstrates the immunosuppressive consequences of contamination with AFB_1_. However, the fact that group C cocks (diet containing 50 g Zn/kg) had significantly lower blood TNF-α concentrations than group B supports the studies that have shown the anti-inflammatory potentials of dietary Zn (Bonaventura et al., 2015). According to Akbari et al., 2018 and Hidayat et al., 2020, Zn is known to control the production of proinflammatory cytokines like TNF-α, suggesting that it may be able to reduce inflammation brought on by AFB_1_. In a similar vein, the addition of 2.50 g VALM/kg to the experimental diet had a beneficial effect on cocks in group D compared to group B birds by considerably lowering their serum TNF-α content. The inclusion of 2.50 g VALM/kg emphasizes the anti-inflammatory potential of VALM in the context of AFB_1_-induced immune response, even though the results were statistically identical to the values shown in the control group. A dose-dependent response, suggesting that higher levels of VALM can enhance its efficacy in lowering TNF-α levels, was recorded among the cocks on diet E. Doubling the VALM inclusion rate to 5.0 g VALM/kg in group E led to a further significant reduction in serum TNF-α concentration compared to groups A, C, and D. The significant reductions observed in IL-1β and TNF-α concentration among the birds in groups D and E could be linked to the chemopreventive properties of *V. amygdalina* with the ability to scavenge free radicals, induce detoxification, inhibit stress response proteins, and interfere with DNA binding activities of some transcription factors (Farombi and Owoeye, 2011). This implies that the biotransformation of AFB_1_ to its toxic epoxide intermediate was reduced by feeding VALM in this study, which, in turn, led to decreased toxicity in the cocks on diets D, and E, respectively.

### Effects of AFB_1_ With or Without *V. amygdalina* and Zn on Serum Anti-Inflammatory Cytokines

This study further highlights the role of AFB_1_ in dampening immune responses (Hatipoglu and Keskin (2022) due the significant depression in serum IL-4 levels among the birds exposed to AFB1 contaminated diet alone compared to the control group (Figure 3). The significant enhancement in serum IL-4 concentration among the cocks on diet C which received AFB_1_-contaminated diets with Zn supplementation compared with group B suggests that Zn supplementation may help counteract the immunosuppressive effects of AFB_1_ and promote IL-4 production (Foster and Samman, 2012). Zn has been shown to play a role in immune regulation, and its supplementation can enhance immune responses (Bonaventura et al., 2015; Zhang et al., 2023). The potential of VALM to ameliorate the immunosuppressive effects of AFB_1_ was clearly highlighted by the inclusion of 2.50 g/kg VALM in group D and 5.00 g/kg VALM in group E which significantly increased the two groups’ serum IL-4 concentrations compared to group B. The tendency of VALM to enhance immune responses, even at higher inclusion rates has been linked with its immunomodulatory properties, and this has suggested the rationale behind the increase in IL-4 levels (Nimiya et al., 2016; Jin et al., 2021). However, the better outcomes (higher concentrations of IL-4 levels) recorded among the birds in group E with higher inclusion of VALM was a testament of the dose-dependency action of VALM inclusion. This suggests that the immunomodulatory effect of VALM on IL-4 levels can be optimized with higher inclusion rates. Importantly, it was observed that the birds in groups D and E had better IL-4 concentrations compared to the birds in group C (AFB_1_ with Zn supplementation), indicating that VALM might have a more significant impact on IL-4 levels compared to inorganic antioxidants like Zn. This result aligns with the idea that natural antioxidants and phytochemicals can exert potent immunomodulatory effects (Nimiya et al., 2016; Jin et al., 2021; Olarotimi et al., 2022). Leukocyte migration and lipid peroxidation reductions are the established mechanisms by which *V. amygdalina* was described to exert its anti-inflammatory effects (Onasanwo et al., 2017).

Further supporting the idea that AFB_1_ exposure has a suppressive effect on the production of cytokines like serum IL-10 is the data shown in Figure 4, which showed a significant decrease in IL-10 concentration among the birds fed a diet containing 1mg/kg AFB_1_ (group B) compared to the control group. Interestingly, the serum IL-10 content was considerably higher in group C compared to group B due to the addition of Zn to the diet that contained 1 mg/kg AFB_1_. This finding demonstrated that Zn supplementation can inhibit the inhibition of IL-10 production caused by AFB_1_. Therefore, through increasing serum IL-10 content, Zn’s immunomodulatory effects had a positive effect on the birds in group C (Bonaventura et al., 2015). When compared to group B, the serum concentration of IL-10 was considerably higher in groups D and E after the addition of 2.50 g/kg VALM and 5.00 g/kg VALM, respectively. This result highlights VALM’s capacity to modulate the immune system in terms of its capacity to promote the production of the anti-inflammatory cytokine such as IL-10. According to Akbari et al. (2018) and Hidayat et al. (2020), the rich phytochemical makeup of VALM, which includes polyphenols and antioxidants, may be a factor in its immunomodulatory effects. Notably, with VALM inclusion, the increase in IL-10 levels also seemed to be dose-dependent. The serum IL-10 content increased noticeably when VALM’s inclusion was doubled, going from 2.50 g/kg in group D to 5.00 g/kg in group E. This emphasizes the opportunity to vary inclusion rates, a characteristic of natural immunomodulators that can be used to enhance the immunomodulatory effects of VALM (Nimiya et al., 2016; Jin et al., 2021).

### Effects of AFB_1_ With or Without *V. amygdalina* and Zn on Serum DNA Damage Biomarkers

In the study, blood 8-OHdG concentrations in cocks exposed to 1 mg/kg AFB_1_ in the diet (group B) were considerably higher than those in the control group. This increase in 8-OHdG levels suggests that exposure to AFB_1_ is causing more oxidative DNA damage. Oxidative stress, which can result in DNA damage, is a known side effect of AFB_1_ (Klaunig et al., 2010; Ayala et al., 2014; Benkerroum, 2020). This result corroborated a previous work by Aleissa et al. (2020), which found that mice given AFB_1_ treatment had higher serum 8-OHdG levels. In addition, this research supported the discovery of Sun et al. (2018) in rabbits fed a diet contaminated with AFB_1_ which had noticeably higher serum 8-OHdG. AFB_1_-exo-8, 9-epoxide (**AFBO**), a poisonous metabolite of AFB_1_, is produced in the liver by the cytochrome P450 enzyme system and cytoplasmic reductase enzymes when AFB_1_ is absorbed into the circulation. When the AFBO binds to DNA, it forms AFB_1_-DNA adducts that lead to mutations and carcinogenesis (Deng et al., 2020; Rajput et al., 2021). But adding Zn to the diet of group C, which contained 1 mg/kg of AFB_1_, had a very beneficial effect. The serum 8-OHdG concentrations were markedly reduced, reaching levels that were similar to those in the control group. The lower levels of oxidative DNA damage seen in this group are most likely due to Zn’s antioxidant capabilities and function in maintaining redox balance (Prasad, 2014; Bonaventura et al., 2015). Zn reduces inflammatory cytokines/molecules in atherosclerosis, such as endothelial cell adhesion molecules and oxidative DNA biomarkers (Bao et al., 2010). Peroxisome proliferator-activated receptor (PPAR-α)-alpha, which is essential for glucose homeostasis, inflammation, and lipoprotein metabolism, is expressed more frequently when Zn is present (Jarosz et al., 2017). A20, a Zn transcription factor, was also upregulated by Zn supplementation, which prevented NF-kB from activating and reduced the synthesis of 8-OHdG (Prasad, 2014). In comparison to group B, the serum 8-OHdG concentrations were significantly reduced by the addition of 2.50 g/kg VALM in group D and 5.00 g/kg VALM in group E. This reveals VALM’s capacity to lessen oxidative DNA damage brought on by AFB1. The protective action of VALM against DNA damage is probably due to its rich phytochemical makeup, which includes antioxidants like polyphenols (Song et al., 2005; Sweeney et al., 2005). Its significant is to note that the drop in 8-OHdG levels seems to be dose-dependent even with VALM inclusion. By increasing VALM from 2.50 g/kg in group D to 5.00 g/kg in group E, the amount of oxidative DNA damage was not only reduced in comparison to group B but also exceeded the levels seen in groups A and C. This shows that increasing the inclusion rate of VALM may increase its capacity to lessen oxidative DNA damage.

According to the findings shown in Figure 6, food exposure to 1 mg/kg AFB_1_ (group B) significantly raised serum NF-kB concentrations in cocks when compared to the control group. This increase in NF-kB activity, which is frequently linked to inflammatory and stress reactions (Park and Hong, 2016; Liu et al., 2017), denotes an NF-kB activation. The group C diet, which contained 1 mg/kg AFB1, benefitted greatly from the addition of Zn. The serum NF-kB concentrations were markedly reduced, reaching values that were similar to those in the control group. The decrease in NF-kB levels seen in this group was probably caused by Zn, which is known for its anti-inflammatory qualities and its involvement in regulating NF-kB activity (Prasad, 2014; Bonaventura et al., 2015). Zn reduces inflammatory cytokines/molecules in atherosclerosis, such as endothelial cell adhesion molecules and oxidative stress biomarkers (Bao et al., 2010). PPAR-α, which is essential for glucose homeostasis, inflammation, and lipoprotein metabolism, is expressed more frequently when Zn is present (Jarosz et al., 2017). In addition, a Zn transcription factor called A20 was upregulated by Zn supplementation, which prevented NF-kB from activating and reduced the generation of inflammatory cytokines (Prasad, 2014). In addition, Zn is excellent at lowering ROS in the body. When 2.50 g/kg VALM was added, group D’s serum NF-kB levels were considerably lower than those of group B. This reveals VALM’s capacity to reduce the NF-kB activation brought on by AFB_1_. VALM’s potent phytochemical makeup, which includes anti-inflammatory and antioxidant substances, probably had a role in its ability to prevent NF-kB activation. It has previously been noted that VALM can suppress, postpone, or destroy malignant cells by triggering apoptosis (Song et al., 2005; Sweeney et al., 2005). Transcriptional factor downregulation has also been connected to the chemopreventive abilities of VALM. Similar to Zn, increased PPAR-α expression from VALM prevents NF-kB activation by producing detrimental cross-talk at the level of nuclear DNA binding (Reiterer et al. 2004; Bao et al. 2010). The fact that the PPAR-α signaling pathway inhibits NF-kB activation shows that VALM affects NF-kB signaling on numerous levels. Importantly, a larger inclusion rate of VALM (5.00 g/kg) in group E appeared to further increase the decline in NF-kB levels. In addition to considerably lowering NF-kB levels in comparison to the other experimental diets, this increased VALM inclusion also suggested the possibility of dose-dependent effects in reducing NF-kB activation.

## CONCLUSIONS

Exposure of chickens to 1.00 mg/kg diet AFB_1_ led to elevation of serum proinflammatory cytokines and suppression of the anti-inflammatory cytokines as well as promoting DNA damage. These results have severe physiological impacts on poultry health and production. It was, however, discovered that both Zn supplementation and VALM inclusion in the diet can effectively counteract the AFB_1_-induced activation of IL-1β, TNF-α, NF-kB, and 8-OHdG. Reduced proinflammatory cytokines activation is crucial for mitigating inflammatory responses and maintaining poultry health. However, the inclusion of 2.50 and 5.0 g/kg diet VALM conferred an ameliorative and immunorestorative effects equal and even better when compared to the effects of dietary Zn.
